# Plasma Cholesterol–Induced Lesion Networks Activated before Regression of Early, Mature, and Advanced Atherosclerosis

**DOI:** 10.1371/journal.pgen.1004201

**Published:** 2014-02-27

**Authors:** Johan L. M. Björkegren, Sara Hägg, Husain A. Talukdar, Hassan Foroughi Asl, Rajeev K. Jain, Cecilia Cedergren, Ming-Mei Shang, Aránzazu Rossignoli, Rabbe Takolander, Olle Melander, Anders Hamsten, Tom Michoel, Josefin Skogsberg

**Affiliations:** 1 Cardiovascular Genomics Group, Division of Vascular Biology, Department of Medical Biochemistry and Biophysics, Karolinska Institutet, Stockholm, Sweden; 2 Cardiovascular Genomics Group, Department of Pathological Anatomy and Forensic Medicine, University of Tartu, Tartu, Estonia; 3 Institute for Genomics and Multi-scale Biology, Icahn School of Medicine at Mount Sinai, New York, New York, United States of America; 4 Department of Medical Epidemiology and Biostatistics, Karolinska Institutet, Stockholm, Sweden; 5 Department of Medical Sciences, Molecular Epidemiology and Science for Life Laboratory, Uppsala University, Uppsala, Sweden; 6 Department of Surgery, Södersjukhuset, Karolinska Institutet, Stockholm, Sweden; 7 Department of Clinical Sciences, Hypertension & Cardiovascular Disease, Clinical Research Centre, Skåne University Hospital, Malmö, Sweden; 8 Atherosclerosis Research Unit, Center for Molecular Medicine, Department of Medicine, Karolinska Institutet, Stockholm, Sweden; 9 Freiburg Institute for Advanced Studies (FRIAS), University of Freiburg, Freiburg, Germany; 10 The Roslin Institute, The University of Edinburgh, Edinburgh, United Kingdom; Stanford University School of Medicine, United States of America

## Abstract

Plasma cholesterol lowering (PCL) slows and sometimes prevents progression of atherosclerosis and may even lead to regression. Little is known about how molecular processes in the atherosclerotic arterial wall respond to PCL and modify responses to atherosclerosis regression. We studied atherosclerosis regression and global gene expression responses to PCL (≥80%) and to atherosclerosis regression itself in early, mature, and advanced lesions. In atherosclerotic aortic wall from *Ldlr^−/−^Apob* ^100/100^ *Mttp* ^flox/flox^Mx1-*Cre* mice, atherosclerosis regressed after PCL regardless of lesion stage. However, near-complete regression was observed only in mice with early lesions; mice with mature and advanced lesions were left with regression-resistant, relatively unstable plaque remnants. Atherosclerosis genes responding to PCL before regression, unlike those responding to the regression itself, were enriched in inherited risk for coronary artery disease and myocardial infarction, indicating causality. Inference of transcription factor (TF) regulatory networks of these PCL-responsive gene sets revealed largely different networks in early, mature, and advanced lesions. In early lesions, *PPARG* was identified as a specific master regulator of the PCL-responsive atherosclerosis TF-regulatory network, whereas in mature and advanced lesions, the specific master regulators were *MLL5* and *SRSF10/XRN2*, respectively. In a THP-1 foam cell model of atherosclerosis regression, siRNA targeting of these master regulators activated the time-point-specific TF-regulatory networks and altered the accumulation of cholesterol esters. We conclude that PCL leads to complete atherosclerosis regression only in mice with early lesions. Identified master regulators and related PCL-responsive TF-regulatory networks will be interesting targets to enhance PCL-mediated regression of mature and advanced atherosclerotic lesions.

## Author Summary

The main underlying cause of heart attacks and strokes is atherosclerosis. One strategy to prevent these often deadly clinical events is therefore either to slow atherosclerosis progression or better, induce regression of atherosclerotic plaques making them more stable. Plasma cholesterol lowering (PCL) is the most efficient way to induce atherosclerosis regression but sometimes fails to do so. In our study, we used a mouse model with elevated LDL cholesterol levels, similar to humans who develop early atherosclerosis, and a genetic switch to lower plasma cholesterol at any time during atherosclerosis progression. In this model, we examined atherosclerosis gene expression and regression in response to PCL at three different stages of atherosclerosis progression. PCL led to complete regression in mice with early lesions but was incomplete in mice with mature and advanced lesions, indicating that early prevention with PCL in individuals with increased risk for heart attack or stroke would be particularly useful. In addition, by inferring PCL-responsive gene networks in early, mature and advanced atherosclerotic lesions, we identified key drivers specific for regression of early (*PPARG*), mature (*MLL5*) and advanced (*SRSF10/XRN2*) atherosclerosis. These key drivers should be interesting therapeutic targets to enhance PCL-mediated regression of atherosclerosis.

## Introduction

Atherosclerosis, primarily in coronary artery disease (CAD) or carotid stenosis, is the main cause of myocardial infarction (MI) and stroke, which together are responsible for more than 50% of deaths worldwide [1]. Although the extent of atherosclerosis in the arterial bed is an unreliable marker of risk for future events, advanced atherosclerotic plaques are present in nearly all cases of MI and in most cases of stroke. It is therefore important to prevent early harmless atherosclerotic lesions from progressing to rupture-prone plaques, and if possible, to induce regression of advanced atherosclerosis into more stable forms [2].

Drugs that lower LDL cholesterol, such as statins, slow atherosclerosis progression and reduce morbidity and mortality from MI and stroke by 30–45% [3]–[5]. More potent statin regimens can even cause atherosclerosis regression [6]–[8] but sometimes have severe side effects. Although statins and lifestyle changes reduce the risk for secondary cardiovascular events [9], mortality from MI and stroke are still increasing [1]. About 10% of persons at increased risk for CAD/MI have elevated plasma cholesterol levels, making them eligible for primary statin treatment. The extent to which plasma cholesterol lowering (PCL) benefits healthy persons who are at increased risk for CAD/MI and have relatively normal plasma LDL-cholesterol levels is unclear [10], [11]. Vulnerable atherosclerotic lesions may respond better to PCL (i.e., leading to regression and more stable plaques) in some cases than in others, depending on inherited genetic and environmental co-factors within the plaque. In part, these factors are likely reflected in gene expression patterns within the plaque [12]. A better understanding of such changes in response to PCL at different stages of plaque development is necessary to define key genes that in themselves or in parallel with PCL help improve atherosclerosis regression.

To effectively study atherosclerosis regression, the use of animal models is required. In earlier studies of atherosclerosis regression, mainly mouse models were used. Among these models were wildtype normolipidemic mice transplanted with atherosclerotic arterial segments from *Apoe^−/−^* mice [13]–[18], *Apoe^−/−^* mice treated with apoE-encoding adenoviral vectors [19], *Ldlr* ^−/−^ mice treated with an microsomal triglyceride transfer protein (MTP) inhibitor [20], and mice that have a plasma lipid profile similar of that of hypercholesterolemia (*Ldlr^−/−^Apob* ^100/100^) and a genetic switch to block hepatic synthesis of lipoproteins and thereby lower plasma lipoproteins (*Mttp* ^flox/flox^Mx1-*Cre*) [21]–[23]. These studies established that PCL leads to atherosclerosis regression. mRNA profiling of atherosclerotic lesions before and after regression led to the identification of several candidate target genes that may mediate atherosclerosis regression after PCL.

However, some important aspects of atherosclerosis regression were overlooked in these studies [13]–[23]. First, the main focus was to identify individual atherosclerosis genes. In contrast, we believe that mRNA profiles are best interpreted by inferring groups of functionally linked genes in disease networks [24], [25]. Another concern relates to the interpretation of molecular changes (reflected by gene expression) in atherosclerotic lesions before, during, and after regression. Typically, atherosclerosis regression candidate genes were identified by comparing mRNA profiles of lesions isolated before and after regression. In our experience, most genes identified in this fashion reflect morphological changes in the atherosclerotic plaque, such as shrinking of the lesion and alterations in the relative cell type composition. Such changes are likely a response to, but not a cause of, atherosclerosis regression. Finally, in a study of gene expression patterns during atherosclerosis progression [12], we observed that the extent of gene expression changes in the atherosclerotic lesions drastically expands and varies as atherosclerosis progresses. Thus, it is highly likely that gene targets to improve PCL-mediated atherosclerosis regression will vary with the stage and severity of the lesions.

In this study, we identified PCL-responsive atherosclerosis genes and their interactions in networks before regression at three stages of atherosclerosis development. We then compared these genes with those responding to the atherosclerosis itself. Specifically, we analyzed the extent, composition, and mRNA profiles of early, mature, and advanced atherosclerotic aortic lesions from *Ldlr^−/−^Apob* ^100/100^ *Mttp* ^flox/flox^Mx1-*Cre* mice [22] immediately before and after Cre-induced PCL and at 10 and 20 weeks after PCL.

## Results

### Study Mice and PCL

To study regression of atherosclerosis at different stages, we lowered plasma lipoprotein levels in *Ldlr^−/−^Apob* ^100/100^ *Mttp* ^flox/flox^Mx1-*Cre* mice by recombining the floxed gene (*Mttp* ^flox/flox^) [26] with polyinosinic-polycytidylic acid (pI-pC) injections after 30, 40, and 50 weeks of atherosclerosis progression (i.e., age of the mice). pI-pC injections in *Mttp* ^wt/wt^Mx1 mice do not affect plasma cholesterol levels or transcriptional activity in the arterial wall [12]. After recombination of microsomal triglyceride transfer protein (*Mttp*), plasma total cholesterol levels were reduced by 80–95%, HDL-cholesterol 50–60% and, triglyceride levels by 40–60%; plasma glucose levels were generally unaffected (Table 1 and Table S1). Plasma cholesterol (both total and HDL) and triglyceride levels were reduced to similar levels in mice with early (week 30), mature (week 40), and advanced (week 50) lesions, and remained at these levels throughout the regression study period (10 and 20 weeks after *Mttp* recombination; Table 1 and Table S1). In littermate *Ldlr^−/−^Apob* ^100/100^ *Mttp* ^flox/flox^Mx1-*Cre* mice injected with PBS (controls) and sacrificed at 20, 30, 40, 50, and 60 weeks, plasma cholesterol and triglyceride levels were unaffected (Table 1). Since *Mttp* recombination primarily affected plasma cholesterol levels (e.g., LDL-cholesterol), we will refer to plasma lipid lowering as PCL.

**Table 1:** Plasma cholesterol, triglyceride and glucose concentrations in the study mice at sacrifice.

	Plasma levels (mg/dl)			
Time point	Before PCL	After PCL for		
		1 week	10 weeks	20 weeks
**20 weeks**				
Cholesterol	220±29			
Triglycerides	85.2±18			
Glucose	407±86			
	(n = 8)			
**30 weeks**				
Cholesterol	254±38	55.0±34***	11.0±5.3***	33.7±26***
Triglycerides	76.5±10	48.6±8.5***	32.7±6.1***	44.3±14***
Glucose	394±78	310±91	335±48*	354±60
	(n = 6)	(n = 6)	(n = 20)	(n = 9)
**40 weeks**				
Cholesterol	264±91	64.9±32***	17.4±12***	24.1±11***
Triglycerides	72.5±10	38.9±13***	38.6±8.6***	62.1±25
Glucose	377±134	293±85	358±69	407±76
	(n = 6)	(n = 5)	(n = 20)	(n = 9)
**50 weeks**				
Cholesterol	226±48	47.9±21***	19.2±9.5***	18.9±4.6***
Triglycerides	97.2±22	51.3±16***	36.8±10***	41.1±5.4***
Glucose	373±64	324±94	336±72	300±120
	(n = 9)	(n = 12)	(n = 25)	(n = 10)
**60 weeks**				
Cholesterol	224±50			
Triglycerides	95.1±47			
Glucose	346±125			
	(n = 9)			

Values are mean ± SD.

*P<0.05,

***P<0.001 vs. before PCL.

### Regression Responses to PCL at Different Stages of Atherosclerosis Progression

**Figure 1:**
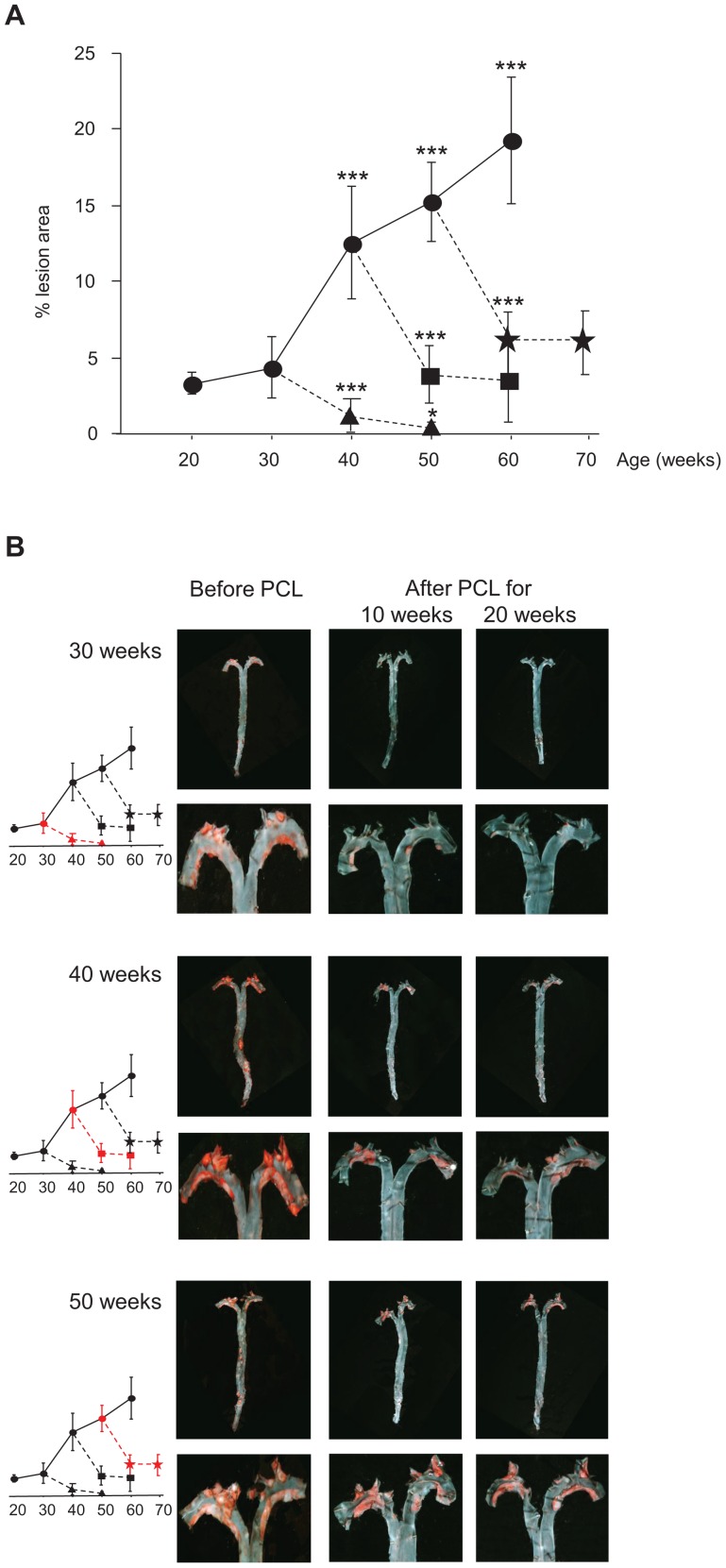
Atherosclerosis progression in *Ldlr^−/−^Apob* ^100/100^ *Mttp* ^flox/flox^ mice and regression in *Ldlr^−/−^Apob* ^100/100^ *Mttp* ^Δ/Δ^ mice. (A) Atherosclerosis progression and regression curves. Values are surface lesion area (mean ± SD), assessed by Sudan IV staining, as a percentage of the total area of pinned-out aortas. n = 4–10 per time point. Lesion development in controls without PCL (•) (*P*<0.001 vs. 30 weeks) and in mice after PCL started at week 30 (▴), 40 (▪), or 50 (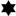). Changes in lesion area between 10 and 20 weeks of low plasma cholesterol were significant only in mice with early lesions (PCL at 30 weeks, *P* = 0.05). *P = 0.05, ***P<0.001. (B) Representative aortic trees (above) with magnified arches (below) stained with Sudan IV before and 10 and 20 weeks after PCL at 30, 40 and 50 weeks. Graphs indicate degree of regression at that PCL time-point (red).

The extent of atherosclerosis progression and regression was assessed by *en face* analysis of the lesion surface area of pinned-out aortic trees stained with Sudan IV. Early lesions in the aortic arch were small and had distinct borders (Figure 1A and 1B). Mature and advanced lesions were substantially larger but still had distinct borders, with small lesions appearing in the ascending aorta.

Atherosclerosis regression occurred at all lesion stages after PCL. In mice with early lesions (30 weeks), PCL led to near-complete regression after 20 weeks (Figure 1A and 1B), from 4.3% of surface area to 0.5% (down by 88%) (*P* = 0.0003). However, in mice with mature lesions (PCL at 40 weeks) and advanced lesions (PCL at 50 weeks), regression was substantial but never complete. During the first 10 weeks of PCL, mature lesions shrank from 12.6% to 4.1% of surface area (*P* = 5×10^−5^) and advanced lesions from 15.2% to 6.2% (*P* = 7×10^−7^). During the last 10 weeks of PCL, however, there was little further regression. Mature lesions shrank from 4.1% to 3.5% of surface area (*P* = 0.6) and advanced lesions from 6.2% to 6.0% (*P* = 0.8) (Figure 1A and 1B). Thus, after 10 weeks of PCL, mature and advanced lesions became resistant to PCL, whereas early lesions continue to regress.

### Plaque Composition after Regression

**Figure 2:**
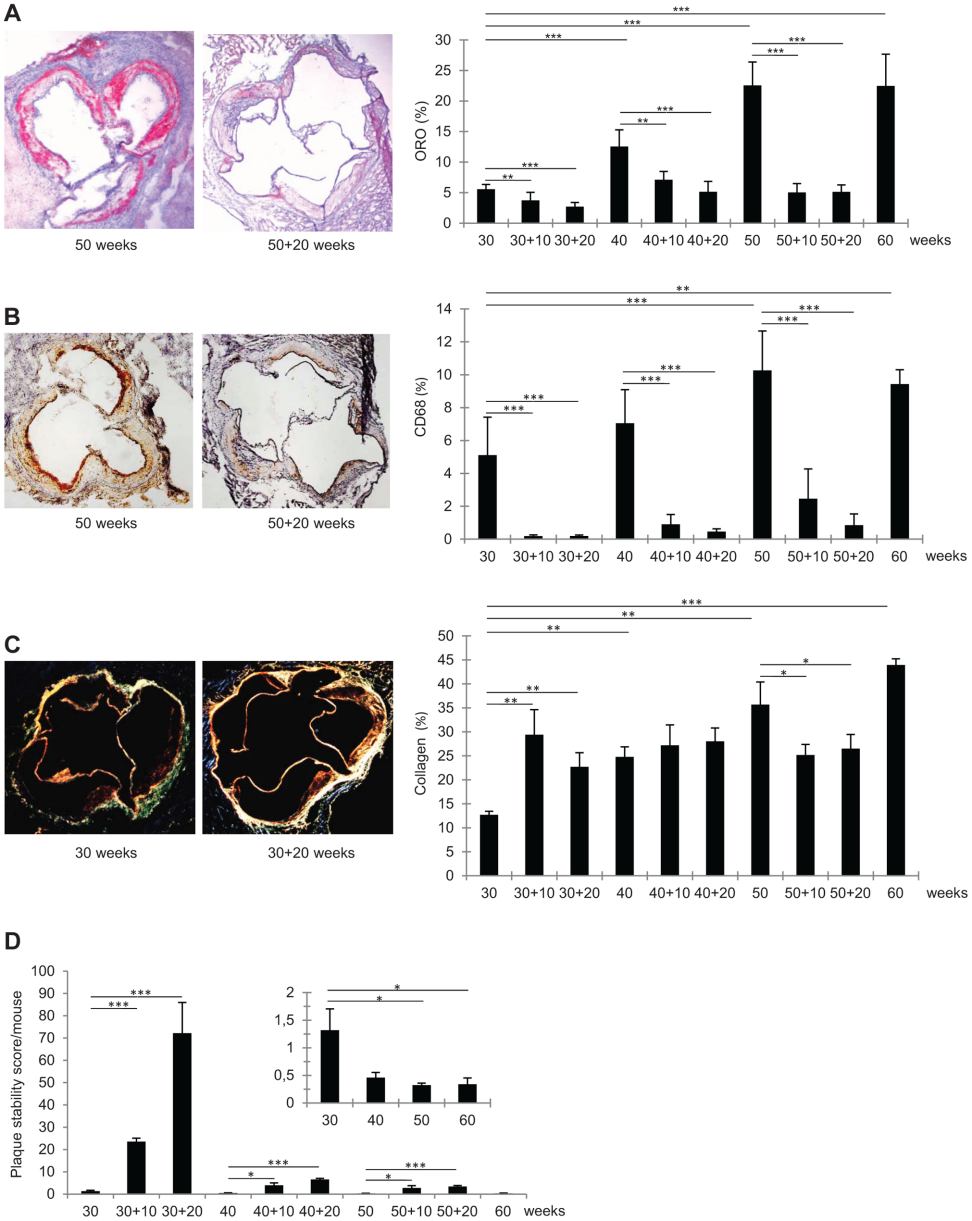
Immunohistochemical characteristics of representative frozen sections of aortic roots from *Ldlr^−/−^Apob* ^100/100^ *Mttp* ^flox/flox^ and *Ldlr^−/−^Apob* ^100/100^ *Mttp* ^Δ/Δ^ mice. (A–C) Average percent stained area of total aortic root area (right) and representative stained aortic roots (left). Bars indicate SD. Original magnification, 50×. *P<0.05, **P<0.01, and ***P<0.001. (A) Oil-Red-O staining (n = 6–9 per group). (B) CD68 staining (n = 5–8 per group). (C) Sirius Red staining (collagen) (n = 3 per group). (D) Mean plaque stability score (arbitrary units). Bars indicate SD. Average plaque stability scores were divided by total extent of plaque burden to assess stability per mouse (not individual plaques). Inset: magnifications of plaque stability score/mouse at 30, 40, 50, and 60 weeks before regression.

Although regression of the extent of atherosclerosis (atherosclerosis burden) increases plaque stability as reflected by the cellular, collagen, and lipid composition of the plaque, compositional changes do not always parallel changes in atherosclerosis burden. Therefore, we compared the histological features of aortic root sections isolated before and 10 and 20 weeks after PCL (Figure 2). Over 20 weeks of regression, the most robust changes in plaque composition were in neutral lipids identified by Oil-Red-O staining (Figure 2A) and in the percentage of lesion macrophages identified by staining for CD68 (Figure 2B). Oil-Red-O staining decreased from 5.6% to 2.7% of surface area in early lesions, from 12.6% to 5.1% in mature lesions, and from 22.6% to 5.1% in advanced lesions (all *P*<0.001). Similarly, the percentage of lesion macrophages decreased from 5.1% to 0.2% in early lesions, from 7.1% to 0.5% in mature lesions, and from 10.3% to 0.8% in advanced lesions (all *P*<0.001). In early lesions, the extensive reduction in the percentage of macrophages (5.1% to 0.2%) was paralleled by near-complete regression after 20 weeks of PCL (Figure 1A). Of note, between weeks 10 and 20 of PCL, the percentage of macrophages in advanced lesions decreased from 2.5% to 0.8% (*P*<0.05) despite no further reduction in the extent of lesions (Figure 1A).

In early lesions, PCL increased the collagen content by 80–130% (*P*<0.01 at 10 and 20 weeks). In mature lesions, the collagen content was unaffected by PCL. In advanced lesions, collagen content decreased by about 30% 10 weeks after PCL (*P*<0.05) and remained at this level at 20 weeks. The pattern of changes in the lesion content of smooth muscle cells (SM22α-positive) was similar to that of collagen content; however, owing to higher variation, none of these changes were statistically significant (data not shown).

### Plaque Stability after Regression

To assess how changes in lesion composition after atherosclerosis regression alter plaque stability, we calculated a stability score: (SM22α+collagen areas)/(CD68+Oil-Red-O areas) [27]. Since this score indicates stability for each plaque and does not consider the total risk for plaque rupture in a given mouse, the plaque score was divided by the total atherosclerosis burden/mouse (i.e., lesion surface area). The resulting stability scores decreased during atherosclerosis progression and increased during regression (Figure 2D).

Early lesions showed the greatest improvement in plaque stability score, which was 17-fold higher after 10 weeks of regression (from 1.3 to 24, *P*<0.001) and 54-fold higher after 20 weeks (1.3 to 72, *P*<0.001). In comparison, after 20 weeks of regression, plaque stability scores had increased only 13-fold in mature lesions (0.5 to 6.6, *P*<0.001) and 11-fold in advanced lesions (0.3 to 3.4, *P*<0.001). Clearly, plaque stability is generally improved by PCL-induced regression; however, in mice with mature and advanced lesions, the baseline score was substantially lower and the improvement much less than in mice with early lesions. Thus, the greatest gain in plaque stability is achieved by PCL in mice with early lesions.

### Gene Expression Profiling of Atherosclerosis Regression

For mRNA profiling studies, PCL was again induced in mice with early (30 weeks), mature (40 weeks), and advanced (50 weeks) lesions. Atherosclerotic aortic arch was isolated for RNA isolation immediately before and after PCL and after 10 weeks of regression. Affymetrix arrays (Mouse Gene 1.0 ST) were used for mRNA profiling.

**Figure 3:**
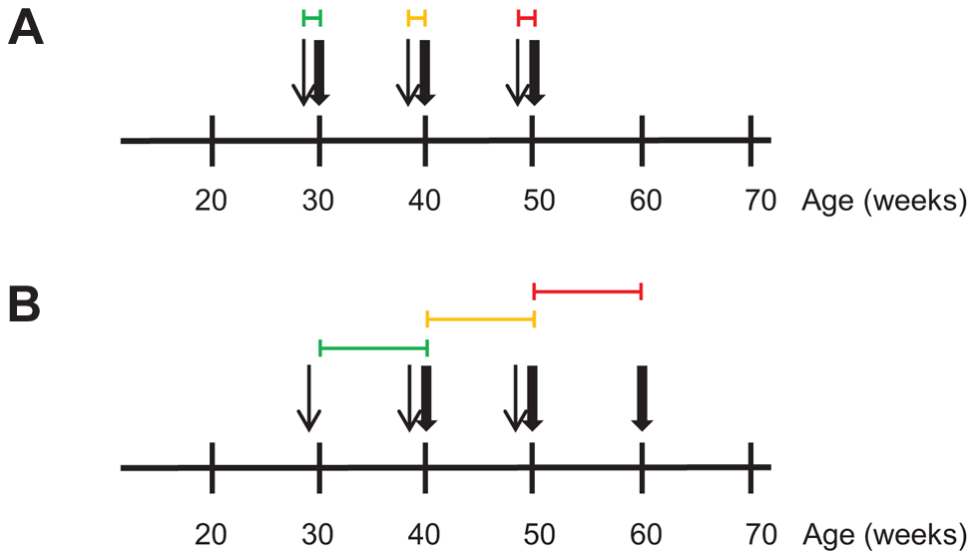
Transcriptional profiling during regression of aortic atherosclerotic lesions in *Ldlr^−/−^Apob* ^100/100^ *Mttp* ^flox/flox^ and *Ldlr^−/−^Apob* ^100/100^ *Mttp* ^Δ/Δ^ mice over time. Differential expression analyses was used to define sets of genes causally and reactively related to atherosclerosis regression in *Ldlr^−/−^Apob* ^100/100^ *Mttp* ^Δ/Δ^ mice. RNA for the transcriptional profiling was isolated from the atherosclerotic aortic arch. Narrow and bold arrows indicate times of PCL and sacrifice, respectively. Colored horizontal lines indicate time frame of transcriptional profiles used for differential expression analysis to define gene sets. Colors indicate when PCL was started: green, 30 weeks; yellow, 40 weeks; red, 50 weeks. (A) To define the PCL-responsive gene sets, we compared transcriptional profiles (4–6 per time point) of PBS-treated, high-cholesterol littermate controls sacrificed at 30, 40 and 50 weeks with those immediately after PCL. (B) To define the regression-reactive gene sets, we compared transcriptional profiles (3–6 per time point) immediately after PCL with those at 10 weeks after PCL (10 per time point).

First, to assess atherosclerotic arterial wall genes that respond to the PCL before atherosclerosis regression (i.e., the PCL-responsive gene set), we compared mRNA profiles immediately before and after PCL (Figure 3A, Tables S2, S3, S4). Since the time between “immediately before and after PCL” is about 1 week, we observed no morphological changes in the lesion composition, including the percentages of different cell types. As a consequence, the PCL-responsive gene sets represent genes with primary changes in their expression levels rather than changes due to alterations in the cellular composition of the plaque.

Next, to identify atherosclerotic arterial wall genes whose expression changed during atherosclerosis regression (i.e., regression-reactive gene sets), we compared mRNA profiles immediately after PCL and after 10 weeks of regression (Figure 3B, Tables S5, S6, S7). In contrast to PCL-responsive gene sets, changes in the expression of many genes in the regression-reactive set likely reflect changes in the cellular composition of the plaque.

**Figure 4:**
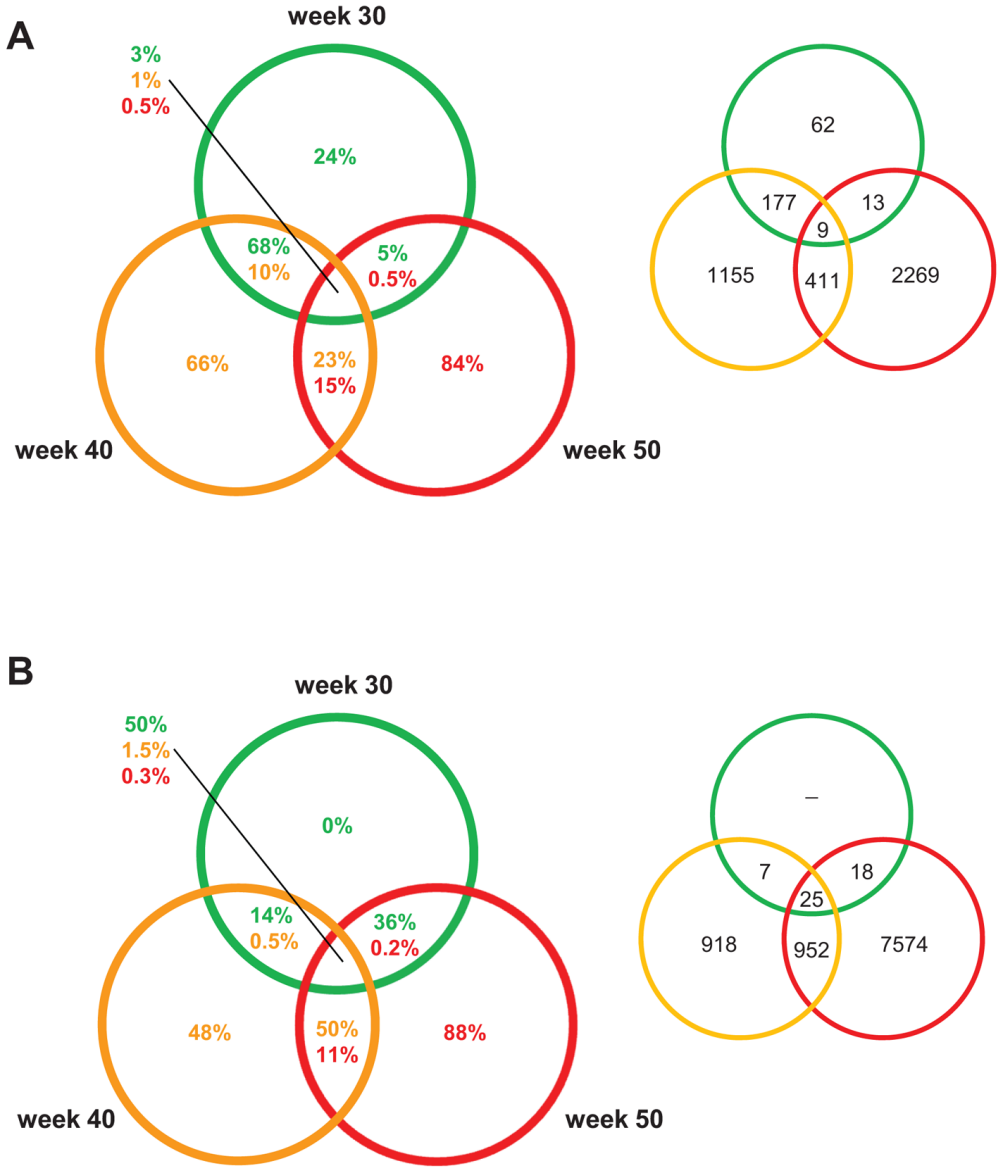
PCL-responsive and regression reactive gene sets of atherosclerosis regression. Venn diagrams showing the percentage/number of differentially expressed genes at 30, 40, and 50 weeks. The colors of the circles indicate when PCL was started: green, 30; yellow, 40 weeks; red, 50 weeks. The percentage in the circles to the left represent the percentage of differentially expressed genes for that section and specific time point. The numbers in circles to the right represent numbers of differentially expressed genes. (A) The PCL-responsive gene sets consist of genes that responded immediately to PCL, initiating regression of early (30 weeks), mature (40 weeks), and advanced (50 weeks) atherosclerosis. (B) The regression-reactive gene sets consist of genes altered in lesions between immediately after PCL and 10 weeks of low plasma cholesterol levels.

As atherosclerotic lesions develop, their molecular complexity increases [12]. So it was not surprising that the number of PCL-responsive genes increased from 261 transcripts (corresponding to 238 mouse genes) in early lesions, to 1752 transcripts (1306 genes) in mature lesions and to 2702 transcripts (2231 genes) in advanced lesions (Figure 3A, Figure 4A, Tables S2, S3, S4). Similarly, the number of regression-reactive genes increased from 50 transcripts (42 genes) in early lesions, to 1902 transcripts (1556 genes) in mature lesions, and up to 8569 transcripts (6273 genes) in advanced lesions (Figure 3B, Figure 4B, Tables S5, S6, S7). These observations suggest that the PCL responses of the atherosclerotic lesions become increasingly complex as atherosclerosis progresses and are mirrored by a greater complexity in the regression response.

Interestingly, the PCL-responsive gene sets were largely unique at each time point/stage of atherosclerosis; the fraction of unique genes was 24%, 66%, and 84% in early lesions, mature, and advanced lesions, respectively (Figure 4A). Of PCL-responsive genes in early lesions, 68% were uniquely shared with PCL-responsive genes in mature lesions but only 5% with advanced lesions (Figure 4A). In contrast, regression-reactive gene sets were more shared between stages of atherosclerosis progression; all regression-reactive genes in early lesions were present in the reactive gene sets of mature and advanced lesions, and 50% of the reactive gene set in mature lesions was present in advanced lesions (Figure 4B). Thus, PCL-responsive atherosclerosis genes largely vary with the stage of atherosclerosis, whereas regression-reactive genes sets expand as atherosclerosis progresses.

### Enrichment of Inherited Risk for CAD/MI of the PCL-Responsive and Regression-Reactive Gene Sets

Genes that are causally linked to (i.e., that drive or protect against) a disease typically harbor DNA variants that affect the risk of developing the disease, whereas genes reacting to a disease typically do not [25]. To examine the causal relationships of the PCL-responsive and regression-reactive gene sets to atherosclerosis regression, we identified single nucleotide polymorphisms (SNPs) affecting the expression of the human orthologs of those genes (Tables S8, S9, S10, S11, S12, S13) and determined the extent to which these expression SNPs (eSNPs) carry more risk for CAD/MI than would be expected by chance. For this purpose, we used a well-established genome-wide association (GWA) study of CAD/MI, MIGen [28].

eSNPs affecting the expression of genes in the PCL-responsive gene sets in early, mature, and advanced atherosclerosis were all risk-enriched compared to 5000 randomly selected equally sized sets of SNPs (early, 2.0-fold, *P* = 3.1×10^−14^; mature, 1.4-fold, *P* = 6.8×10^−4^; advanced, 1.5-fold, *P* = 1.3×10^−6^). In contrast, eSNPs affecting the expression of genes in the regression-reactive gene sets were not (early/mature/advanced, <1.1-fold, *P*>0.05). These results support the notion that PCL-responsive genes are causally linked to regression of atherosclerosis and that regression-responsive genes are, as hypothesized, secondary.

### PCL-Responsive TF-Regulatory Gene Network of Early Atherosclerosis

From the standpoint of understanding genes that drive atherosclerosis regression, genes that respond acutely to PCL and are risk-enriched for CAD/MI—that is, the PCL-responsive gene sets of early, mature, and advanced atherosclerosis—were considered the most interesting.

**Figure 5:**
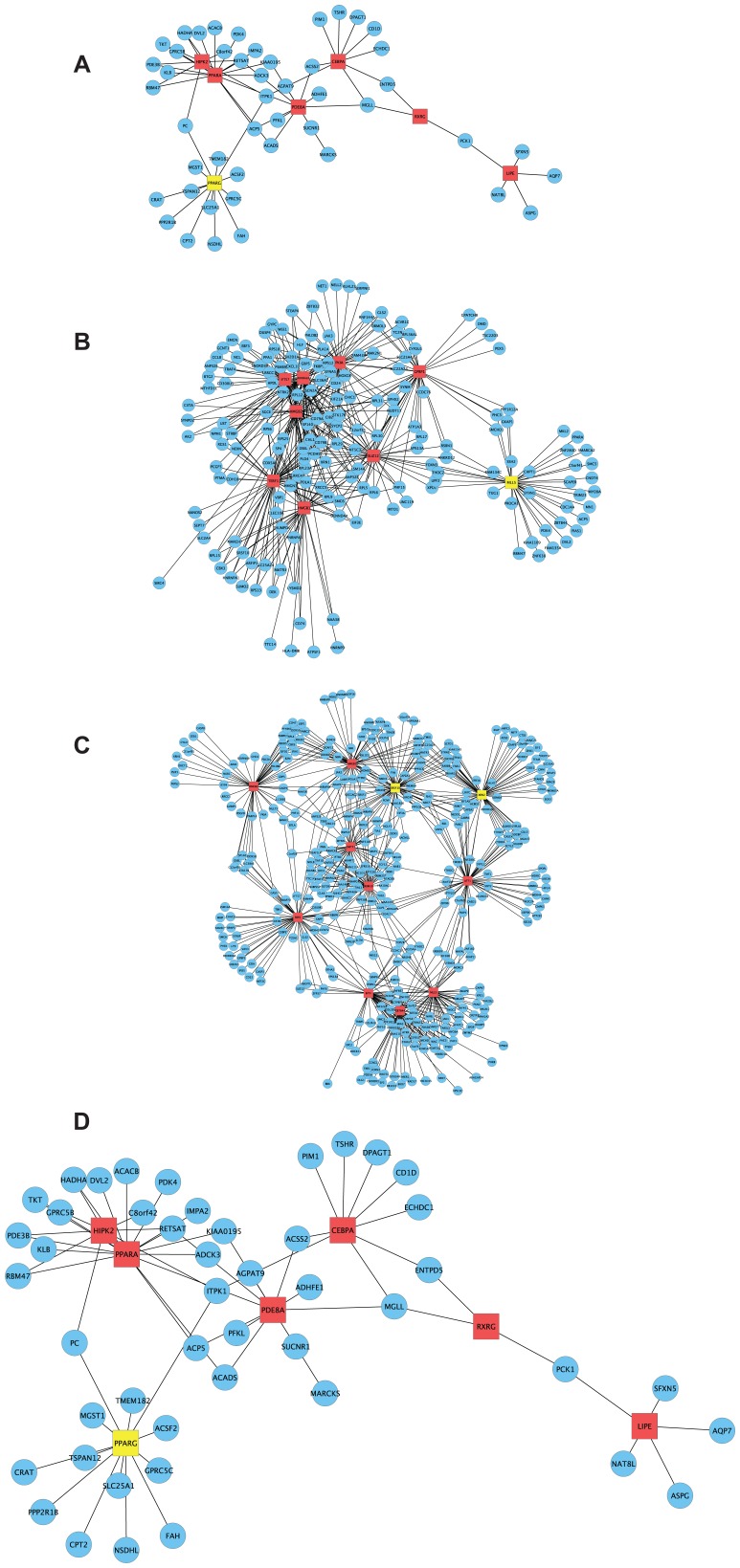
CAD-patient macrophage TF-regulatory coexpression networks of PCL-responsive genes linked to atherosclerosis regression. To learn more about functional interactions of the PCL-responsive gene sets using human orthologs, we used macrophage mRNA profiles (n = 38) from patients with CAD [29] to infer TF-regulatory gene networks. Red square nodes are TFs. Yellow square nodes are specific master regulatory TFs (Table 4): *PPARG* for the network in early lesions (30 weeks) and *MLL5* for the network in mature lesions (40 weeks) and *SRSF10* and *XRN2* for the network in advanced lesions (50 weeks). Edges are connections between TFs and their first neighbor. (A) At 30 weeks, 53 genes of 215 human orthologs belonged to the TF-regulatory network (*P*<0.0051), in which the most connected TFs (master regulators) were *PPARA* (17 edges) and *PPARG* (13 edges) (Table 2). The TF-regulatory network of PCL-responsive atherosclerosis regression genes at 30 weeks is magnified in (D) to show all nodes. (B) At 40 weeks, 185 genes of 1087 human orthologs in the causal gene set belonged to the TF-regulatory network (*P*<0.0013). The most connected TFs were *HMGB2*, *ADORA2A*, and *TERF1*, with 61, 59 and 55 edges, respectively (Table 2). (C) At 50 weeks, 379 genes of 1865 human orthologs in the causal gene set belonged to the TF-regulatory network (*P*<0.00042), in which the most connected TFs were *SRSF10*, *XRN2*, and *HMGB1*, with 71, 67 and 62 edges, respectively (Table 2). (D) A magnification of the TF regulatory network of PCL-responsive genes at week 30, shown in (A).

According to GO analysis of the PCL-responsive gene set of early lesions (n = 261), the top molecular and cellular function was *lipid metabolism* and the top disease category was *connective tissue disorder* (Table S14). Next, to investigate the connectivity of the human orthologs of the PCL-responsive genes of early lesions (n = 215), we inferred the TF-regulatory gene network by using mRNA profiles from blood macrophages of CAD patients [29]. Fifty-three of 215 human orthologs belonged to the TF-regulatory network (Figure 5A, *P*<0.0051, Table S8). Peroxisome proliferator-activated receptor alpha and gamma (*PPARA*, *PPARG*) were master regulators (highly connected genes) in this network, with 17 and 13 edges, respectively (Table 2).

**Table 2:** Top hubs in the causal TF-regulatory co-expression networks inferred in human macrophages.

Gene symbol	Gene name	Network Connections		
Top hubs week 30				
*PPARA*	PPAR alpha	17		
*PPARG*	PPAR gamma	13		
*PDE8A*	Phosphodiesterase 8A	12		
*CEBPA*	CCAAT/enhancer-binding protein alpha	9		
*HIPK2*	Homeodomain interacting protein kinase	9		
Top hubs week 40				
*HMGB2*	High mobility group box 2	61		
*ADORA2A*	Adenosine A2a receptor	59		
*TERF1*	Telomeric repeat binding factor 1	55		
*PKIA*	Protein kinase inhibitor alpha	48		
*HMGB1*	High mobility group box 1	44		
Top hubs week 50				
*SRSF10*	Serine/arginine-rich splicing factor 10	71		
*XRN2*	5′-3′ exoribonuclease 2	67		
*HMGB1*	High mobility group box 1	62		
*ATF1*	Activating transcription factor 1	60		
*BMI1*	BMI1 polycomb ring finger oncogene	59		
Common hubs weeks 30 and 40				
None				
Common hubs weeks 40 and 50		Week 40	Week 50	
* HMGB1*	High mobility group box 1	44	62	
* HMGB2*	High mobility group box 2	61	54	
* MLL5*	Myeloid/lymphoid or mixed-lineage leukemia 5	38	55	

TF, transcription factor. Common hubs >5 connections.

To validate the PCL-responsive TF-regulatory gene network in early lesions, including its key master regulators, we used a THP-1 regression model. In brief, THP-1 cells were differentiated into macrophages *in vitro* and incubated with acetylated-LDL (Ac-LDL) to form foam cells. The cells were then treated with siRNA (to silence the key master regulators) or mock treated (controls) and examined for effects on the expression levels of network genes and cholesterol-ester (CE) accumulation. CE levels were assessed from lipids in the THP-1 foam cells after siRNA silencing of the master regulator and compared CE levels in mock-treated cells. If CE accumulation increased, the master regulator was judged to promote atherosclerosis regression. If CE accumulation decreased, the master regulator was judged to prevent regression. To assess gene expression in THP-1 foam cells, RNA isolated after silencing was analyzed with an Agilent Human Custom Gene Expression Microarray; the degree of silencing of master regulators was assessed by RT-PCR.

When *PPARG* was silenced, 28% (15/53) of the PCL-responsive network genes in early atherosclerosis were affected (down- or up-regulated at a false-discovery rate (FDR)<0.1; Table 3 and Tables S8 and S17). A hypergeometric test (Methods) showed that the effects of silencing *PPARG* were specific to the 53 genes in the TF-regulatory gene network of early lesions (*P* = 0.020, Table 4). In the THP-1 foam cell model, CE accumulation increased by 12% (*P* = 0.008) after silencing of *PPARG* (Table 5).

**Table 3:** Number of affected genes its respective network when the master regulatory TF were silenced with siRNA.

	Number of affected genes		
Gene symbol	30 weeks^a^	40 weeks^b^	50 weeks^c^
*PPARA*	1	-	-
*PPARG*	15	-	-
*ADORA2A*	-	58	-
*HMGB1*	-	3	4
*HMGB2*	-	2	0
*MLL5*	-	15	4
*SRSF10*	-	-	71
*XRN2*	-	-	55

a 53 network genes (FDR<0.1);

b 185 network genes (FDR<0.1);

c 379 network genes (FDR<0.05);

TF, transcription factor; -, not applicable.

**Table 4:** Network specificity of the key master regulators using hypergeometric testing.

	Hypergeometric *P* value		
Gene symbol	30 weeks	40 weeks	50 weeks
*PPARA*	0.079	0.27	1.00
***PPARG***	**0.020**	0.15	1.00
*HMGB2*	1.00	1.00	1.00
*ADORA2A*	0.94	0.95	0.41
*HMGB1*	0.39	0.21	0.47
***MLL5***	0.76	**0.0059**	1.00
***SRSF10***	0.95	0.89	**0.035**
***XRN2***	0.91	0.58	**0.040**

**Table 5:** Effects of siRNA inhibition of the network top hubs on cholesterol-ester accumulation in a THP-1 foam cell model.

Gene symbol	Control (relative CE levels)	siRNA knock (relative CE levels)	CE content (% relative control)	*P* value
*PPARG*	100±9.0	112±13	+12	0.008
*MLL5*	100±17	121±20	+21	0.01
*SRSF10*	100±8.4	82.8±9.2	−17	0.0008
*XRN2*	100±12	85.2±17	−15	0.003

CE, cholesterol ester.

### PCL-Responsive TF Regulatory Gene Network of Mature Atherosclerosis

According to GO analysis of the PCL-responsive gene set (n = 1752) of mature lesions, the top molecular and cellular function was *lipid metabolism* (Table S15) and the top disease categories were *connective tissue disorder* and *metabolic disease* (Table S15). Next, we investigated the connectivity of these genes by inferring the TF-regulatory gene network, again using the mRNA profiles from blood macrophages of CAD patients [29]. Of 1087 human orthologs (corresponding to 1306 mouse genes, Table S9), 185 were part of the inferred TF-regulatory gene network (*P*<0.0013, Figure 5B, Table S9, Figure S1). The master regulators of this network were high mobility group box 2 (*HMGB2*), adenosine A2a receptor (*ADORA2A*), telomeric repeat binding factor 1 (*TERF1*), and mixed lineage leukemia 5 (*MLL5*), with 61, 59, 55 and 38 connections, respectively (Table 2).

To validate the PCL-responsive TF-regulatory gene network and its key master regulators in mature lesions, we again used a THP-1 foam cell regression model. After silencing of *ADORA2A*, 31% (58/185) of the mature network genes were affected; after silencing of both *ADORA2A* and *MLL5*, 36% (67/185) were affected (FDR<0.1, Table 3, Table S9 and Table S17). *MLL5* was the only key master regulator that was specific for the mature atherosclerosis network according to the hypergeometric test (*P* = 0.0059, Table 4). After *MLL5* silencing, CE accumulation in the THP-1 foam cell model increased 21% (*P* = 0.01, Table 5).

### PCL-Responsive TF Regulatory Gene Network of Advanced Atherosclerosis

According to GO analysis of the PCL-responsive gene set (n = 2702) in advanced lesions, the top molecular and cellular functions were *protein synthesis* and *degradation* (Table S16) and the top disease categories were *immunological disease* and *cardiovascular disease* (Table S16). Next, we investigated the connectivity of these genes by inferring the TF-regulatory gene network, using the same mRNA profiles from blood macrophages of CAD patients [29] (Figure 5C). Of 1865 human orthologs (corresponding to 2231 mouse genes, Table S10), 379 were part of the inferred regulatory gene network (*P*<0.00042, Figure 5C, Table S10, Figure S2). The master regulators in this network were serine/arginine-rich splicing factor 10 (*SRSF10*), 5′-3′-exoribonuclease 2 (*XRN2*), and *HMGB1*, with 71, 67, and 62 connections, respectively (Table 2).

In the THP-1 foam cell regression model, silencing of both *SRSF10* and *XRN2* affected 22% (83/379) of the advanced network genes (FDR<0.05, Table 3, Table S10 and Table S17). Silencing of *SRSF10* and *XRN2* individually affected 19% and 15%, respectively, of the advanced lesion network genes. Both *SRSF10* and *XRN2* were specific master regulators for the advanced atherosclerosis network (*P* = 0.035, *P* = 0.040, respectively, Table 4). In the THP-1 foam cell model, CE accumulation decreased by 17% (*P* = 0.0008) after silencing of *SRSF10* and by 15% (*P* = 0.003) after silencing of *XRN2* (Table 5).

## Discussion

PCL decreases the risk for clinical complications of atherosclerosis, but individual responses vary, from slowing or preventing further progression to inducing regression. This study of a mouse model with human-like plasma lipoprotein profile and advanced atherosclerotic lesions showed that atherosclerosis regression occurs regardless of the lesion stage at which PCL is induced. However, as lesions progress, they become increasingly resistant to PCL. In mice with early lesions, PCL led to a complete regression and nearly healthy arteries (plaque stability score >70 after 20 weeks of regression). In mice with mature lesions, the regression was incomplete, leaving plaque remnants that were substantially smaller but relatively instable (stability score <10). And in mice with advanced lesions, the plaque remnants were even less stable (stability score <5). Thus, if early atherosclerosis in humans is equally sensitive to plasma cholesterol levels, patients at increased risk for CAD and MI would benefit greatly from PCL while their lesions are still in the early stage.

The increasing resistance to PCL as atherosclerotic plaques progress suggests that specific molecular processes in atherosclerosis regulate PCL sensitivity and thus the response to atherosclerosis regression. We therefore performed mRNA-profiling immediately before and after PCL to identify PCL-responsive atherosclerosis genes and examined their interplay in TF-regulatory gene networks. Consistent with the differences in plaque sensitivity to PCL, plasma cholesterol-responsive genes in the atherosclerotic arterial wall were largely different in early, mature, and advanced lesions. In early lesions, we identified *PPARG* as a specific master regulator of other PCL-responsive genes that collectively led to near-complete regression. In mature and advanced plaques, we identified nonspecific master regulators (affecting both mature and advanced PCL-responsive genes in THP-1 foam cells), such as *ADORA2A*, *HMGB1*, *HMGB2*, and *TERF1*, as well as specific master regulators of partial regression in mature lesions (*MLL5*) and advanced lesions (*SRSF10* and *XRN2*). In validation studies in THP-1 foam cells, siRNA targeting individual master regulators either decreased (*SRSF10*, *XRN2*) or increased (*PPARG* and *MLL5*) CE accumulation. These genes are plausible targets to improve PCL-mediated regression of mature and advanced atherosclerosis.

In studies to validate the inherited risk-enrichment [25] of the PCL-responsive and regression-reactive gene sets, we found that only PCL-responsive genes were enriched with inherited risk for CAD/MI (>1.4-fold, *P*<6.8×10^−4^). The causal gene set of early atherosclerosis was especially risk enriched (2.0-fold, *P* = 3.1×10^−14^), perhaps indicating that the causal gene set of early lesions precedes those of mature and advanced lesions and has a more important role in carrying inherited risk. We [12], [25] and others [24] have shown that molecular processes with key roles in disease have at least some degree of risk enrichment. However, genes affected by DNA variants (i.e., eSNPs) might be disease relevant despite not necessarily carrying inherited risk. Thus, some regression-reactive genes are likely important for atherosclerosis regression despite their lack of enrichment in inherited risk of CAD/MI. The lack of risk enrichment in the regression-reactive gene sets does not imply that every gene or pathway in these sets is irrelevant for atherosclerosis regression. For example, the regression-reactive gene sets included many genes in the transendothelial migration of leukocytes (TEML) pathway that are thought to be important in regression [19].

Interestingly, master regulatory genes did not harbor any disease-associated eSNPs according to the MIGen GWA dataset [28], although many other PCL-responsive network genes did. What is responsible for this difference? One possibility is that SNPs or mutations in genes that encode key transcription regulatory proteins (i.e., master regulators) often are deleterious and therefore are effectively eliminated by natural selection from the gene pool [30]. In support of this notion, disease risk loci identified by GWA studies so far have not yet identified key master regulators of lipid metabolism in CAD, like SREBPs, PPARs and LXR [31], [32]. Instead, genes in lipid metabolism that have been identified by GWA studies, such as PCSK9, ABCG5 and ABCG8 [31], [32], are, to our understanding, important modifiers but not master regulators.

Although some regression-reactive genes may contribute to atherosclerosis regression, they did not respond to PCL. Responsiveness to PCL, we believe, is key to the atherosclerosis regression response. Interestingly, *PPARG* was identified as a PCL-responsive master regulator of the TF-regulatory network of early lesions. Recently a study of the same mouse model we used showed that treatment with pioglitazone (a PPARG agonist) in addition to PCL improved the inflammatory profile of CD68 cells [21]. Thus, the PPARG agonist modified the response of the atherosclerotic arterial wall to PCL. Our validation of stage-specific master regulators in the foam cell model of regression suggests that *MLL5*, *SRSF10*, and *XRN2* will be useful targets for improving atherosclerosis regression after PCL in individuals with mature or even advanced lesions. Studies targeting these genes either genetically or with drugs in parallel to PCL are warranted.

Atherosclerosis regression after PCL has been investigated in several mouse models [13]–[23], [33] and the results prompted debate about the mechanisms of regression. According to a leading theory, regression is caused by increased macrophage emigration from the plaque [13], [16], [18], [33]. Another study suggested that the key mechanism is suppressed migration of leukocytes to the arterial wall [19]. The notion that monocyte migration is a key process in regression is supported by our transcriptional profiling data and immunohistological characteristics of atherosclerosis regression (loss of CD68-positive cells and decrease in Oil-Red-O staining). In contrast, we found that expression of chemokine (C-C motif) receptor 7 (*Ccr7*) and liver X receptor alpha (*Lxr*) was downregulated in response to atherosclerosis regression, not upregulated (not shown). These findings suggest that deactivation of TEML pathway genes, rather than increased emigration of macrophages, is more essential for atherosclerosis regression. In relation to the PCL-responsive gene sets, the TEML pathway may be a key event but is activated further downstream, since a majority of these genes did not respond to PCL. In addition, our findings clearly indicate that atherosclerosis regression is too complex to be explained by changes in TEML activity alone.

Besides migrating and emigrating, macrophages within the plaque also proliferate, affecting plaque size [34]. Specifically, at a high turnover rate, lesion macrophages can be replenished by local proliferation rather than de novo influx of monocytes [34]. How macrophage proliferation rate is affected (if at all) by PCL at different stages of atherosclerosis progression is unknown but is certainly of interest for future studies. Our findings show that enhancing atherosclerosis regression after PCL will require targeting regulatory genes at the top of the regulatory hierarchy (e.g., master regulators) rather than individual effector genes (e.g., *Ccr7* and *Lxr*) or specific pathways, such as TEML or cell proliferation.

Interestingly, even though mature and advanced plaque remnants became increasingly resistant to PCL-mediated regression, the number of CD68-positive cells decreased between weeks 10 and 20 of PCL in all plaques. This was most clear in early lesions, leading to near-complete regression. However, in mature plaques, and particularly in advanced plaques, despite a substantial decrease in CD68-positive cells between weeks 10 and 20, lesion size was mainly unaffected. Over this period, the plaque stability scores improved, but it is difficult to interpret the underlying biology of these changes. One plausible explanation is that macrophage death (necrosis) is responsible for the reduction of CD68-positive cells in advanced plaques; the lack of change in plaque size would reflect a larger necrotic core despite the lower number of macrophages. Another plausible explanation is that there are fewer but larger macrophages; however, since there could be many CD68 proteins/macrophage, this explanation seems less likely. Regardless, the plaque stability scores clearly coincided with the degree of regression, indicating that when all regression-induced compositional changes to the plaques were jointly considered (i.e., CD68-positive cells, lipid accumulation, collagenous matrix, and vascular smooth muscle cells), the remnants of mature and advanced plaques remained relative unstable after both 10 and 20 weeks of regression.

The mouse model used in this study is, in our opinion, the most relevant model for investigating atherosclerosis regression and associated changes in gene expression after PCL. First, these mice develop advanced atherosclerotic lesions on a normal chow diet and have a plasma lipid profile very similar to that of patients with familial hypercholesterolemia, who are highly susceptible to CAD [22], [35]. Second, the high cholesterol levels in these mice can effectively be lowered after inducing expression of Mx1-*Cre* in the liver upon pI-pC treatments resulting in the recombination of *Mttp*. This can be achieved at any time during lesion progression without affecting vitality [22]. Of note, however, unlike human atherosclerotic plaques those in mice rarely rupture [36].

There are other ways to induce atherosclerosis regression than by lowering “bad” LDL-cholesterol levels (the main form of “PCL” in the present study). It is also possible to overexpress ApoA1, the major apolipoprotein of HDL-cholesterol (“good” cholesterol), to enhance reverse cholesterol transport from the plaques to the liver. This strategy is motivated by the fact that plasma levels of HDL-cholesterol and ApoA1 in humans correlate inversely and independently with coronary heart disease [37], [38]. Furthermore, in *Apoe* ^−/−^ mice, infusion of recombinant ApoA1 lowers lipid and macrophage levels in the plaque [39]. However, adenoviral transfer of ApoA1 into *Ldlr* ^−/−^ mice has had inconsistent results [40]–[42]. In one study, preexisting atherosclerotic lesions regressed after ApoA1 gene transfer [41]; however, in other studies, regression was not detected, but progression was slowed, and plaques in the aortic root had fewer macrophages and more collagen content [40], [42]. Yet, the consensus is that increased levels HDL-cholesterol on top of LDL-cholesterol lowering could have additive effects on atherosclerosis regression and result in a more stable phenotype [39], [42], [43]. However, the plasma HDL-cholesterol levels in our study mice were reduced after PCL, which has also been shown by others [22], indicating that reverse cholesterol transport might not play a major role in the regression of atherosclerosis in our model.

Investigating transcriptional responses in the whole atherosclerotic arterial wall is not entirely trivial. As alluded to already, gene expression changes in the arterial wall may reflect gene activation (i.e., changes in the cellular mRNA concentrations) or changes in the cellular composition of the lesion. However, in our experience, it is vital to have data from the entire lesion, as many molecular processes are intermixed and depend on gene activity across cell types. The ideal situation would be to have mRNA profiles of single cell types (e.g., macrophages) together with the total mRNA profile of the same arterial wall. Unfortunately, this is not feasible in most instances. Another challenge of cell-type-specific mRNA profiles is to accurately distinguish different cell types before RNA isolation. This is especially difficult in atherosclerosis where, for example, smooth muscle cells sometimes change their phenotype and become macrophage-like cells [44]. In sum, as atherosclerotic lesions develop, different cell types become increasingly similar, sharing many phenotypes—a fact that favors the use of mRNA profiles from the entire lesion rather than from isolated cell types. However, since gene networks inferred from whole-lesion mRNA data will be incomplete and have missing nodes, it is essential to evaluate these networks in appropriate cell models of atherosclerosis, such as the THP-1 foam cell model we used in this study.

We also reasoned that the transcriptional profiles of blood macrophages isolated from CAD patients [29] would be most relevant for establishing the wiring diagram of the identified PCL-responsive mouse genes in TF-regulatory gene networks. This choice allowed us to understand mouse genes from the perspective of pathophysiological processes in humans [25], [29], [45] and to use a human foam cell culture model to validate the TF-regulatory networks, including their hubs (i.e., master regulators).

In summary, in this study we identified comprehensive compendiums of atherosclerosis regression genes and discovered that the sensitivity of atherosclerosis to PCL depends on the stage of lesion progression. Our findings provide insight into PCL-responsive genes upstream of atherosclerosis regression and challenge aspects of our understanding of this clinically important event [13], [15]–[23], [33]. In particular, our findings emphasize the need to determine how PCL-responsive genes collectively initiate downstream regression mechanisms, such as the TEML pathway and possibly macrophage emigration and proliferation. Master regulators such as *PPARG*, *MLL5*, *XRN2*, and *SRSF10* merit further study to determine the extent to which combinations of these genes can be activated or deactivated to achieve complete regression of advanced atherosclerotic lesions, with or without parallel PCL regimens.

## Materials and Methods

### Ethics Statement

The use of human samples [29] in this study was approved by the Ethics Committee of Karolinska University Hospital. All patients gave written, informed consent. The animal studies were approved by Stockholm's Norra Djurförsöksetiska nämnd, Sweden.

### Mouse Model

*Ldlr* ^−/−^ *Apob* ^100/100^ *Mttp* ^flox/flox^Mx1-*Cre* mice have a plasma lipoprotein profile which resembles that of familial hypercholesterolemia and causes rapid atherosclerosis progression [22]. For *Mttp* deletion, mice were injected with 125 µl of pI-pC (1 µg/µl; Invivogen) every other day for 6 days to induce *Cre* expression and thereby *Mttp* recombination (*Mttp* ^Δ/Δ^). The mice were sacrificed 1, 10, or 20 weeks after *Mttp* depletion. Littermate controls received PBS (*Mttp* ^flox/flox^). The study mice were backcrossed 5 times to C57BL/6 mice (<5% 129/SvJae and >95% C57BL/6), housed in a pathogen-free barrier facility (12-hour light/12-hour dark cycle), and fed rodent chow containing 4% fat. Plasma cholesterol (total and HDL) and triglyceride concentrations in fasting blood samples were determined with colorimetric assays (Infinity cholesterol/triglyceride kits; Thermo Scientific and HDL quantification colorimetric kit; BioVision), and plasma glucose levels with Precision Xtra (MediScience).

### *En Face* Analysis and Histology

Aortas were pinned out flat on black wax surface as described [46], stained with Sudan IV, photographed with a Nikon SMZ1000 microscope, and analyzed with Easy Image Analysis 2000 software (Tekno Optik, Sweden). Lesion area was calculated as the percentage of the entire aortic surface between the aortic root and the iliac bifurcation. Aortic roots were isolated, immediately frozen in liquid nitrogen, embedded in OCT compound (Histolab, Sweden), cut into 10-µm sections, and stained with hematoxylin and Oil-Red-O (Sigma-Aldrich) for neutral lipids [47] or Picrosirius Red (Sigma-Aldrich) for collagen as described [48]. Other sections were incubated first with rat anti-mouse CD68 antibody (Serotec) or rabbit anti-mouse SM22α (Abcam) overnight at 4°C and then with biotinylated secondary anti-rat IgG or anti-rabbit IgG antibodies (Vector Laboratories) and counterstained with hematoxylin (Sigma-Aldrich). Biotin emission was developed with diaminobenzidine (Vector Laboratories). Except for sections stained for collagen, which were photographed with a Leica DMRD microscope and a Leica DC480 color video camera, sections were photographed with an Apotome microscope (Carl Zeiss) and quantified with an AxioGraphic station (Carl Zeiss) at 50× magnification. For *en face* analysis, we examined aortas from 44 *Ldlr* ^−/−^ *Apob* ^100/100^ *Mttp* ^flox/flox^ mice (n = 12, 12, 8, 8, and 4 for weeks 20, 30, 40, 50, and 60, respectively) and 51 *Ldlr* ^−/−^ *Apob* ^100/100^ *Mttp* ^Δ/Δ^ mice (early lesions: n = 10 after 10 weeks and n = 6 after 20 weeks of PCL; mature lesions: n = 8 after both 10 and 20 weeks of PCL; advanced lesions: n = 9 and 10 after 10 and 20 weeks of PCL). The plaque stability score for each mouse was calculated as (SM22α+collagen)/(CD68+Oil-Red-O) % areas [27] and normalized to plaque burden (lesion surface area). Missing data points (n = 19, 16%) were imputed with PROC MI in SAS version 9.3. The statistical significance of differences between time points was determined with two-tailed *t* tests.

### RNA Isolation and Global Gene Expression Profiling of Mouse Aortic Arch

Aortas were perfused with PBS and then with RNAlater (Qiagen), and the aortic arch (third rib to aortic root) was removed (to get RNA from the most atherosclerotic part of the aorta) and homogenized with FastPrep (Qbiogene). Total RNA was isolated with an RNeasy Mini-kit with a DNAse I treatment step (Qiagen). RNA quality was assessed with a Bioanalyzer 2100 (Agilent Technologies), and RNA quantity with NanoDrop (Thermo Scientific). Global mRNA expression profiles were generated with Mouse Gene 1.0 ST arrays (Affymetrix) according to the manufacturer's protocol. In brief, amplified and biotinylated cRNA was generated from 100 ng of high-quality RNA with the GeneChip WT Sense Target Labeling and Control Reagents kit (No. 900652, Affymetrix). The arrays were hybridized in a GeneChip Hybridization Oven 640, further processed with a Fluidics Station 450, scanned with a GeneArray Scanner 3000 7G, and analyzed with GeneChip Operational Software 2.0. For global gene expression profiling, 18 *Ldlr* ^−/−^ *Apob* ^100/100^ *Mttp* ^flox/flox^ control mice (n = 6, 6, and 6 at week 30, 40, and 50, respectively) and 48 *Ldlr* ^−/−^ *Apob* ^100/100^ *Mttp* ^Δ/Δ^ mice (early lesions: n = 6 immediately after PCL and n = 10 after PCL for 10 weeks; mature lesions: n = 6 immediately after PCL and n = 10 after PCL for 10 weeks; advanced lesions: n = 6 immediately after PCL and n = 10 after PCL for 10 weeks) were used.

### Analyses of Mouse Gene Expression Data

All samples were randomized and run simultaneously on the arrays at the Bioinformatics and Expression Analysis Core Facility at the Karolinska Institutet. Global mRNA expression data were pre-processed with the three-step Robust Multichip Average [49] procedure (background correction, quantile normalization, and summarization). No batch effects were detected that needed to be adjusted for. However, when comparing within groups of samples a few samples were considered outliers and excluded from further analyses. Groups of samples were compared by using differential expression (FDR<0.30) [50]. The FDR level was selected partly on the basis of sensitivity analysis [51] to capture an adequately large portion of the true-positive genes for the following downstream analysis. Mathematica 7.0 and 8.0 or R 2.9.2 “package: affy” was used for all calculations. Selected probe sets were annotated with NetAffx (Affymetrix) and DAVID [52], [53]. Ingenuity Systems Pathway Analysis (IPA, www.ingenuity.com) was used to for functional analyses of sets of differentially expressed genes. The top bio-function categories—*molecular and cellular functions* and *disease and disorders—* were used.

### Global Gene Expression Profiles of Human Macrophages

Primary human monocytes from carotid endarterectomy patients [29] were isolated from 80 ml of EDTA-treated blood by density-gradient centrifugation and Ficoll-Paque Plus (Amersham Biosciences). The monocyte-enriched layer was collected, washed, and plated in RPMI 1640 (Gibco-Invitrogen) supplemented with penicillin (100 U/ml) and streptomycin (100 µg/ml) (PEST) and 10% human AB serum (Sigma-Aldrich) in six-well plates (BD Bioscience). The next day, nonadherent cells were removed, and the remaining monocyte/macrophage-enriched cells were given fresh RPMI 1640 medium supplemented as described above. After 7 days, total RNA from adherent macrophages was isolated with the RNeasy Mini-kit (Qiagen). Thirty-eight mRNA expression profiles were generated with custom microarrays (Affymetrix GeneChip HuRSTA-2a520709). The Robust Multichip Average algorithm in Affymetrix Power Tools (version 1.14.2) was used for background subtraction, normalization, and summarizing of raw microarray data.

### CAD/MI Risk Enrichment Analyses Using the MIGen Genome-Wide Association Dataset

If the gene activity of the identified mouse gene sets (PCL-responsive and regression-reactive) are important for atherosclerosis progression/regression (rather than being reactive markers of disease development), eSNPs of the identified gene sets could be enriched for CAD/MI risk. An eSNP indicates a functional relationship between the SNP and the expression of the identified gene (within 1 Mb upstream and downstream of transcription start site) [29], [54]. To investigate this, we first identified human orthologs of the differentially expressed mouse genes using HUGO Gene Nomenclature Committee's (HGNC) Human and Mouse Orthologous Gene Nomenclature and National Center for Biotechnology Information's (NCBI) HomoloGene (Tables S8, S9, S10, S11, S12, S13). Enrichment of eSNPs with CAD/MI risk was determined with GWA data from MIGen [28]. eSNPs were identified from global genotype data (n = 156) and mRNA expression profiles of *in vitro* differentiated blood macrophages [29]. eSNPs were expanded with SNPs in strong linkage disequilibrium (r^2^>0.9) within 200 kb of the eSNPs using HapMap. The expanded SNP sets for the causal gene sets consisted of 168, 511, and 1170 SNPs and the reactive gene sets of 17, 276, and 1057 SNPs for 30, 40, and 50 weeks, respectively; overlapping SNPs between causal and reactive SNP sets were removed from the reactive SNP sets. A total of 5000 random samples of SNPs were used to determine whether the expanded SNP set was more likely to be associated with CAD/MI than randomly selected sets with the same characteristics (i.e., equal number of SNPs, chromosomal distribution, and minor allele frequency >5%). Finally, fold enrichment in risk was calculated as the ratio between the relative number of significant SNPs (*P*<0.05) in the expanded SNP set and the relative number of significant SNPs (*P*<0.05) in the random sets. MATLAB R2011a was used for all computations.

### Regulatory Gene Network Reconstructions

TF-regulatory co-expression networks were reconstructed from global mRNA expression profiles from blood macrophages [29]. Regulatory gene networks were inferred from human homologs of causal mouse genes using the context likelihood of relatedness (CLR) method with Pearson correlation [55], [56]. The CLR method computes the significance of a given regulator-target similarity score for a gene regulatory network. In brief, by using Pearson correlation, co-expression similarity between all gene pairs was computed and stored in a matrix, M [55]. Next, background corrections using positive z-scores were computed for each entry for M, considering both row and column values. Then, the joint likelihood of pairwise z-scores for each M was assessed [56]. TF-regulatory interactions used for the networks in early, mature, and advanced lesions had *P* values of <0.0051, <0.0013, and <0.00042, respectively, corresponding to the 50% most probable interactions in each network. CLR with Pearson correlation was implemented in C++. For each time point, the 10 most connected TFs are shown in the visualization of the networks with Cytoscape 2.8.2 [57].

### siRNA Perturbation of THP-1 Macrophages Incubated with Ac-LDL

Human THP-1 monocytes were plated at 5×10^5^ cells/well in six-well culture dishes (Becton Dickinson) containing 10% fetal calf serum (FCS)-RPMI-1640 supplemented with PEST. The cells were incubated with PMA (50 ng/mL) (Sigma-Aldrich) for 72 hours to induce differentiation into macrophages and with Ac-LDL (50 µg/mL) for 48 hours to generate foam cells. Thereafter, for each master regulator, cells were transfected with siRNA (one at a time) (Ambion, Life Technologies, Table S17) using Lipofectamine 2000 as recommended by the manufacturer (Invitrogen), in medium without FCS, PEST, or PMA. Forty-eight hours after siRNA transfection, cells were examined for effects on the expression of network genes (see section Gene Expression Measurements and Hypergeometric Testing for Network Specificity) and CE accumulation (see section Lipid and Protein Measurements).

Ac-LDL was prepared as described [58]. The samples were then dialyzed against PBS at 4°C. Ac-LDL protein concentration was determined by the Bradford method. LDL was isolated from plasma of healthy donors by sequential ultracentrifugation [59].

### Gene Expression Measurements and Hypergeometric Testing for Network Specificity

For differential expression analyses and to determine the degree of silencing by siRNA (Table S17), total RNA was isolated from the targeted THP-1 foam cells with the RNeasy Mini-kit (Qiagen). The concentration was determined by NanoDrop (Thermo Scientific). The degree of siRNA silencing was determined by TaqMan analyses (Table S17). cDNA was synthesized from 0.4 µg of total RNA with Superscript III (Invitrogen). Diluted cDNA was amplified by real-time PCR with 1×TaqMan universal PCR master mix (Applied Biosystems) according to the manufacturer's protocol. Assay-On-Demand kits with corresponding primers and probes from Applied Biosystems were used (Table S17); samples were normalized with the comparative Ct method. mRNA expression profiles of targeted THP-1 foam cells were generated with Agilent Human Custom Gene Expression Microarray 8×15K, containing network genes (556 unique genes from the 30, 40, and 50 week networks) and TEML genes (116 TEML genes from DAVID (two of these 116 genes are present among the network genes) as well as three macrophage emigration genes (*CCR7*, *LXR*, and *NTN1*), for a total 673 of unique genes (spotted in duplicate), according to the manufacturer's instructions. The R package “limma” was used to normalize the Agilent array data and to identify differentially expressed genes (FDR<0.1) with the Benjamini-Hochberg procedure.

The probability that a master regulator was specific for its time-point network rather than expected by chance was calculated by using hypergeometric distribution *P* values as follows:
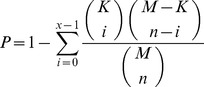
When sampling *X* genes of *M* genes (*M* = 673 array genes), what is the probability (*P*) that *x* or more of these genes belong to a time-point-specific network *K* (*K* = 53, 185, or 379 genes for the 30-, 40-, or 50-week network, respectively), shared by *n* of the M genes (Table 4).

### Lipid and Protein Measurements

Lipids from siRNA-targeted THP-1-derived foam cells were isolated by extraction with hexane/isopropanol (3∶2) at room temperature for 1 hour and then with 0.5 ml of chloroform for 15 min [60]. The lipids were dried and resuspended in isopropanol with 1% Triton-X-100 (Sigma-Aldrich). The lipid content of the foam cells was determined by enzymatic assays using the Infinity kit for total cholesterol (Thermo Scientific) and a kit for free cholesterol (Wako Chemicals). After lipid extraction, proteins were extracted from the same wells by incubation with 0.5 M sodium hydroxide for 5 hours at 37°C. Protein concentration was determined by the Bradford method. CE accumulation in the targeted THP-1 foam cells was calculated as total cholesterol – free cholesterol/protein concentration, relative to control.

## Supporting Information

Figure S1 A magnification of the TF regulatory network of PCL-responsive genes at week 40, shown in Figure 5B. Reds square nodes are TFs. The yellow square node show the specific master regulatory TF for the mature regression network, *MLL5*. Edges are connections between TFs and their first neighbor.
(EPS)

Figure S2 A magnification of the TF regulatory network of PCL-responsive genes at week 50, shown in Figure 5C. Reds square nodes are TFs. Yellow square node show the specific master regulatory TFs for the advanced regression network, *SRSF10* and *XRN2*. Edges are connections between TFs and their first neighbor.
(EPS)

Table S1 Plasma HDL-cholesterol concentrations in the study mice at sacrifice.
(XLSX)

Table S2 PCL-responsive genes at week 30.
(XLSX)

Table S3 PCL-responsive genes at week 40.
(XLSX)

Table S4 PCL-responsive genes at week 50.
(XLSX)

Table S5 Regression reactive genes at week 30.
(XLSX)

Table S6 Regression reactive genes at week 40.
(XLSX)

Table S7 Regression reactive genes at week 50.
(XLSX)

Table S8 Human orthologs for PCL-responsive mouse genes and network connections at week 30.
(XLSX)

Table S9 Human orthologs for PCL-responsive mouse genes and network connections at week 40.
(XLSX)

Table S10 Human orthologs for PCL-responsive mouse genes and network connections at week 50.
(XLSX)

Table S11 Human orthologs for Regression reactive mouse genes at week 30.
(XLSX)

Table S12 Human orthologs for Regression reactive mouse genes at week 40.
(XLSX)

Table S13 Human orthologs for Regression reactive mouse genes at week 50.
(XLSX)

Table S14 Functional enrichment using Ingenuity category Top Bio Function for PCL-responsive genes at week 30.
(XLSX)

Table S15 Functional enrichment using Ingenuity category Top Bio Function for PCL-responsive genes at week 40.
(XLSX)

Table S16 Functional enrichment using Ingenuity category Top Bio Function for PCL-responsive genes at week 50.
(XLSX)

Table S17 siRNA and TaqMan assays and the degree of siRNA inhibition.
(XLSX)

## References

[1] Global Atlas on Cardiovascular Disease Prevention and Control (Mendis S, Puska P, Norrving B, eds). Geneva: World Health Organization, 2011.

[2] Brown MS, Goldstein JL (2006) Biomedicine. Lowering LDL–not only how low, but how long? Science 311: 1721–1723. 16556829 10.1126/science.1125884

[3] Brown BG, Zhao XQ, Sacco DE, Albers JJ (1993) Lipid lowering and plaque regression. New insights into prevention of plaque disruption and clinical events in coronary disease. Circulation 87: 1781–1791. 8504494 10.1161/01.cir.87.6.1781

[4] LaRosa JC, Grundy SM, Waters DD, Shear C, Barter P, et al (2005) Intensive lipid lowering with atorvastatin in patients with stable coronary disease. N Engl J Med 352: 1425–1435. 15755765 10.1056/NEJMoa050461

[5] Ong HT (2005) The statin studies: from targeting hypercholesterolaemia to targeting the high-risk patient. QJM 98: 599–614. 16006501 10.1093/qjmed/hci093

[6] Nissen SE, Nicholls SJ, Sipahi I, Libby P, Raichlen JS, et al (2006) Effect of very high-intensity statin therapy on regression of coronary atherosclerosis: the ASTEROID trial. JAMA 295: 1556–1565. 16533939 10.1001/jama.295.13.jpc60002

[7] Crouse JR 3rd, Raichlen JS, Riley WA, Evans GW, Palmer MK, et al (2007) Effect of rosuvastatin on progression of carotid intima-media thickness in low-risk individuals with subclinical atherosclerosis: the METEOR Trial. JAMA 297: 1344–1353. 17384434 10.1001/jama.297.12.1344

[8] Grines CL (2006) Transcatheter cardiovascular therapeutics annual meeting. J Interv Cardiol 19: 183–210. 16650251 10.1111/j.1540-8183.2006.130_1.x

[9] Chan AW, Bhatt DL, Chew DP, Quinn MJ, Moliterno DJ, et al (2002) Early and sustained survival benefit associated with statin therapy at the time of percutaneous coronary intervention. Circulation 105: 691–696. 11839623 10.1161/hc0602.103586

[10] Taylor F, Ward K, Moore TH, Burke M, Davey Smith G, et al (2011) Statins for the primary prevention of cardiovascular disease. Cochrane Database Syst Rev CD004816. 21249663 10.1002/14651858.CD004816.pub4 PMC4164175

[11] Taylor F, Huffman MD, Macedo AF, Moore TH, Burke M, et al (2013) Statins for the primary prevention of cardiovascular disease. Cochrane Database Syst Rev 1: CD004816. 23440795 10.1002/14651858.CD004816.pub5 PMC6481400

[12] Skogsberg J, Lundstrom J, Kovacs A, Nilsson R, Noori P, et al (2008) Transcriptional profiling uncovers a network of cholesterol-responsive atherosclerosis target genes. PLoS Genet 4: e1000036. 18369455 10.1371/journal.pgen.1000036 PMC2265530

[13] Feig JE, Pineda-Torra I, Sanson M, Bradley MN, Vengrenyuk Y, et al (2010) LXR promotes the maximal egress of monocyte-derived cells from mouse aortic plaques during atherosclerosis regression. J Clin Invest 120: 4415–4424. 21041949 10.1172/JCI38911 PMC2993578

[14] Reis ED, Li J, Fayad ZA, Rong JX, Hansoty D, et al (2001) Dramatic remodeling of advanced atherosclerotic plaques of the apolipoprotein E-deficient mouse in a novel transplantation model. J Vasc Surg 34: 541–547. 11533609 10.1067/mva.2001.115963

[15] Trogan E, Fayad ZA, Itskovich VV, Aguinaldo JG, Mani V, et al (2004) Serial studies of mouse atherosclerosis by in vivo magnetic resonance imaging detect lesion regression after correction of dyslipidemia. Arterioscler Thromb Vasc Biol 24: 1714–1719. 15256400 10.1161/01.ATV.0000139313.69015.1c

[16] Trogan E, Feig JE, Dogan S, Rothblat GH, Angeli V, et al (2006) Gene expression changes in foam cells and the role of chemokine receptor CCR7 during atherosclerosis regression in ApoE-deficient mice. Proc Natl Acad Sci U S A 103: 3781–3786. 16537455 10.1073/pnas.0511043103 PMC1450154

[17] Feig JE, Vengrenyuk Y, Reiser V, Wu C, Statnikov A, et al (2012) Regression of atherosclerosis is characterized by broad changes in the plaque macrophage transcriptome. PLoS One 7: e39790. 22761902 10.1371/journal.pone.0039790 PMC3384622

[18] Llodra J, Angeli V, Liu J, Trogan E, Fisher EA, et al (2004) Emigration of monocyte-derived cells from atherosclerotic lesions characterizes regressive, but not progressive, plaques. Proc Natl Acad Sci U S A 101: 11779–11784. 15280540 10.1073/pnas.0403259101 PMC511052

[19] Potteaux S, Gautier EL, Hutchison SB, van Rooijen N, Rader DJ, et al (2011) Suppressed monocyte recruitment drives macrophage removal from atherosclerotic plaques of Apoe^−^/^−^ mice during disease regression. J Clin Invest 121: 2025–2036. 21505265 10.1172/JCI43802 PMC3083793

[20] Hewing B, Parathath S, Mai CK, Fiel MI, Guo L, et al (2013) Rapid regression of atherosclerosis with MTP inhibitor treatment. Atherosclerosis 227: 125–129. 23332773 10.1016/j.atherosclerosis.2012.12.026 PMC4047651

[21] Feig JE, Parathath S, Rong JX, Mick SL, Vengrenyuk Y, et al (2011) Reversal of hyperlipidemia with a genetic switch favorably affects the content and inflammatory state of macrophages in atherosclerotic plaques. Circulation 123: 989–998. 21339485 10.1161/CIRCULATIONAHA.110.984146 PMC3131163

[22] Lieu HD, Withycombe SK, Walker Q, Rong JX, Walzem RL, et al (2003) Eliminating atherogenesis in mice by switching off hepatic lipoprotein secretion. Circulation 107: 1315–1321. 12628954 10.1161/01.cir.0000054781.50889.0c

[23] Parathath S, Grauer L, Huang LS, Sanson M, Distel E, et al (2011) Diabetes adversely affects macrophages during atherosclerotic plaque regression in mice. Diabetes 60: 1759–1769. 21562077 10.2337/db10-0778 PMC3114401

[24] Schadt EE (2009) Molecular networks as sensors and drivers of common human diseases. Nature 461: 218–223. 19741703 10.1038/nature08454

[25] Schadt EE, Bjorkegren JL (2012) NEW: network-enabled wisdom in biology, medicine, and health care. Sci Transl Med 4: 115rv111. 10.1126/scitranslmed.3002132 22218693

[26] Raabe M, Veniant MM, Sullivan MA, Zlot CH, Bjorkegren J, et al (1999) Analysis of the role of microsomal triglyceride transfer protein in the liver of tissue-specific knockout mice. J Clin Invest 103: 1287–1298. 10225972 10.1172/JCI6576 PMC408359

[27] Ni W, Egashira K, Kitamoto S, Kataoka C, Koyanagi M, et al (2001) New anti-monocyte chemoattractant protein-1 gene therapy attenuates atherosclerosis in apolipoprotein E-knockout mice. Circulation 103: 2096–2101. 11319201 10.1161/01.cir.103.16.2096

[28] Kathiresan S, Voight BF, Purcell S, Musunuru K, Ardissino D, et al (2009) Genome-wide association of early-onset myocardial infarction with single nucleotide polymorphisms and copy number variants. Nat Genet 41: 334–341. 19198609 10.1038/ng.327 PMC2681011

[29] Hagg S, Skogsberg J, Lundstrom J, Noori P, Nilsson R, et al (2009) Multi-organ expression profiling uncovers a gene module in coronary artery disease involving transendothelial migration of leukocytes and LIM domain binding 2: the Stockholm Atherosclerosis Gene Expression (STAGE) study. PLoS Genet 5: e1000754. 19997623 10.1371/journal.pgen.1000754 PMC2780352

[30] Ramensky V, Bork P, Sunyaev S (2002) Human non-synonymous SNPs: server and survey. Nucleic Acids Res 30: 3894–3900. 12202775 10.1093/nar/gkf493 PMC137415

[31] Holdt LM, Teupser D (2013) From genotype to phenotype in human atherosclerosis–recent findings. Curr Opin Lipidol 24: 410–418. 24005217 10.1097/MOL.0b013e3283654e7c PMC3814939

[32] Roberts R, Stewart AF (2012) Genes and coronary artery disease: where are we? J Am Coll Cardiol 60: 1715–1721. 23040572 10.1016/j.jacc.2011.12.062

[33] Feig JE, Shang Y, Rotllan N, Vengrenyuk Y, Wu C, et al (2011) Statins promote the regression of atherosclerosis via activation of the CCR7-dependent emigration pathway in macrophages. PLoS One 6: e28534. 22163030 10.1371/journal.pone.0028534 PMC3232231

[34] Robbins CS, Hilgendorf I, Weber GF, Theurl I, Iwamoto Y, et al (2013) Local proliferation dominates lesional macrophage accumulation in atherosclerosis. Nat Med 19: 1166–1172. 23933982 10.1038/nm.3258 PMC3769444

[35] Grundy SM (1986) Cholesterol and coronary heart disease. A new era. JAMA 256: 2849–2858. 3534335

[36] Bentzon JF, Falk E (2010) Atherosclerotic lesions in mouse and man: is it the same disease? Curr Opin Lipidol 21: 434–440. 20683327 10.1097/MOL.0b013e32833ded6a

[37] Goldbourt U, Yaari S, Medalie JH (1997) Isolated low HDL cholesterol as a risk factor for coronary heart disease mortality. A 21-year follow-up of 8000 men. Arterioscler Thromb Vasc Biol 17: 107–113. 9012644 10.1161/01.atv.17.1.107

[38] Gordon DJ, Rifkind BM (1989) High-density lipoprotein–the clinical implications of recent studies. N Engl J Med 321: 1311–1316. 2677733 10.1056/NEJM198911093211907

[39] Shah PK, Yano J, Reyes O, Chyu KY, Kaul S, et al (2001) High-dose recombinant apolipoprotein A-I(milano) mobilizes tissue cholesterol and rapidly reduces plaque lipid and macrophage content in apolipoprotein e-deficient mice. Potential implications for acute plaque stabilization. Circulation 103: 3047–3050. 11425766 10.1161/hc2501.092494

[40] Li R, Chao H, Ko KW, Cormier S, Dieker C, et al (2011) Gene therapy targeting LDL cholesterol but not HDL cholesterol induces regression of advanced atherosclerosis in a mouse model of familial hypercholesterolemia. J Genet Syndr Gene Ther 2: 106. 23106034 10.4172/2157-7412.1000106 PMC3480198

[41] Tangirala RK, Tsukamoto K, Chun SH, Usher D, Pure E, et al (1999) Regression of atherosclerosis induced by liver-directed gene transfer of apolipoprotein A-I in mice. Circulation 100: 1816–1822. 10534470 10.1161/01.cir.100.17.1816

[42] Van Craeyveld E, Gordts SC, Nefyodova E, Jacobs F, De Geest B (2011) Regression and stabilization of advanced murine atherosclerotic lesions: a comparison of LDL lowering and HDL raising gene transfer strategies. J Mol Med (Berl) 89: 555–567. 21249329 10.1007/s00109-011-0722-x PMC3098380

[43] Feig JE, Rong JX, Shamir R, Sanson M, Vengrenyuk Y, et al (2011) HDL promotes rapid atherosclerosis regression in mice and alters inflammatory properties of plaque monocyte-derived cells. Proc Natl Acad Sci U S A 108: 7166–7171. 21482781 10.1073/pnas.1016086108 PMC3084076

[44] Gomez D, Owens GK (2012) Smooth muscle cell phenotypic switching in atherosclerosis. Cardiovasc Res 95: 156–164. 22406749 10.1093/cvr/cvs115 PMC3388816

[45] Tegner J, Skogsberg J, Bjorkegren J (2007) Thematic review series: systems biology approaches to metabolic and cardiovascular disorders. Multi-organ whole-genome measurements and reverse engineering to uncover gene networks underlying complex traits. J Lipid Res 48: 267–277. 17142807 10.1194/jlr.R600030-JLR200

[46] Veniant MM, Sullivan MA, Kim SK, Ambroziak P, Chu A, et al (2000) Defining the atherogenicity of large and small lipoproteins containing apolipoprotein B100. J Clin Invest 106: 1501–1510. 11120757 10.1172/JCI10695 PMC387257

[47] Stotz E, Schenk EA, Churukian C, Willis C (1986) Oil red O: comparison of staining quality and chemical components as determined by thin layer chromatography. Stain Technol 61: 187–190. 2425463 10.3109/10520298609110730

[48] Wagsater D, Zhu C, Bjorkegren J, Skogsberg J, Eriksson P (2011) MMP-2 and MMP-9 are prominent matrix metalloproteinases during atherosclerosis development in the Ldlr^−/−^Apob^100/100^ mouse. Int J Mol Med 28: 247–253. 21567073 10.3892/ijmm.2011.693

[49] Irizarry RA, Bolstad BM, Collin F, Cope LM, Hobbs B, et al (2003) Summaries of Affymetrix GeneChip probe level data. Nucleic Acids Res 31: e15. 12582260 10.1093/nar/gng015 PMC150247

[50] Efron B, Tibshirani R, Storey J, Tusher V (2001) Empirical Bayes analysis of a microarray experiment. J Am Stat Assoc 96: 1151–1160.

[51] Pawitan Y, Michiels S, Koscielny S, Gusnanto A, Ploner A (2005) False discovery rate, sensitivity and sample size for microarray studies. Bioinformatics 21: 3017–3024. 15840707 10.1093/bioinformatics/bti448

[52] Huang da W, Sherman BT, Lempicki RA (2009) Systematic and integrative analysis of large gene lists using DAVID bioinformatics resources. Nat Protoc 4: 44–57. 19131956 10.1038/nprot.2008.211

[53] Huang da W, Sherman BT, Lempicki RA (2009) Bioinformatics enrichment tools: paths toward the comprehensive functional analysis of large gene lists. Nucleic Acids Res 37: 1–13. 19033363 10.1093/nar/gkn923 PMC2615629

[54] Schadt EE, Molony C, Chudin E, Hao K, Yang X, et al (2008) Mapping the genetic architecture of gene expression in human liver. PLoS Biol 6: e107. 18462017 10.1371/journal.pbio.0060107 PMC2365981

[55] Faith JJ, Hayete B, Thaden JT, Mogno I, Wierzbowski J, et al (2007) Large-scale mapping and validation of Escherichia coli transcriptional regulation from a compendium of expression profiles. PLoS Biol 5: e8. 17214507 10.1371/journal.pbio.0050008 PMC1764438

[56] Madar A, Greenfield A, Vanden-Eijnden E, Bonneau R (2010) DREAM3: network inference using dynamic context likelihood of relatedness and the inferelator. PLoS One 5: e9803. 20339551 10.1371/journal.pone.0009803 PMC2842436

[57] Shannon P, Markiel A, Ozier O, Baliga NS, Wang JT, et al (2003) Cytoscape: a software environment for integrated models of biomolecular interaction networks. Genome Res 13: 2498–2504. 14597658 10.1101/gr.1239303 PMC403769

[58] Basu SK, Goldstein JL, Anderson GW, Brown MS (1976) Degradation of cationized low density lipoprotein and regulation of cholesterol metabolism in homozygous familial hypercholesterolemia fibroblasts. Proc Natl Acad Sci U S A 73: 3178–3182. 184464 10.1073/pnas.73.9.3178 PMC430973

[59] Redgrave TG, Carlson LA (1979) Changes in plasma very low density and low density lipoprotein content, composition, and size after a fatty meal in normo- and hypertriglyceridemic man. J Lipid Res 20: 217–229. 220359

[60] Christoffersen C, Nielsen LB, Axler O, Andersson A, Johnsen AH, et al (2006) Isolation and characterization of human apolipoprotein M-containing lipoproteins. J Lipid Res 47: 1833–1843. 16682745 10.1194/jlr.M600055-JLR200

